# Ultrasound findings of the elbow posterior fat pad in children with radial head subluxation

**DOI:** 10.1186/2036-7902-6-S1-A28

**Published:** 2014-01-31

**Authors:** JE Rabiner, H Khine, JR Avner, JW Tsung

**Affiliations:** 1 Department of Pediatrics, Division of Pediatric Emergency Medicine, Children’s Hospital at Montefiore / Albert Einstein College of Medicine, Bronx, NY, USA; 2 Department of Emergency Medicine, Division of Pediatric Emergency Medicine, Mount Sinai Medical Center / Mount Sinai School of Medicine, New York, NY, USA

## Background

In young children with a non-mobile elbow, it can be difficult to differentiate radial head subluxation (RHS) from elbow fracture by history and examination alone. Data demonstrate that an elevated posterior fat pad (PFP) or lipohemarthrosis (LH) of the PFP on ultrasound is highly sensitive for fracture at the elbow, but it is not known whether these findings are present in children with RHS.

## Objective

To determine if elbow ultrasound findings of the PFP are present in patients with the diagnosis of RHS.

## Patients and methods

This was a prospective study of children presenting to an urban pediatric emergency department diagnosed clinically with RHS. Physicians received a one-hour training session on musculoskeletal ultrasound including the elbow. Prior to performing reduction for RHS, physicians performed a brief, point-of-care elbow ultrasound using a high-frequency linear transducer probe in both longitudinal and transverse views to evaluate for PFP elevation and LH. Successful clinical reduction with spontaneous movement of the injured extremity served as the gold standard for RHS. Clinical telephone follow up was performed to ascertain outcomes.

## Results

42 patients were enrolled with a mean age of 22.3 (± 11.8) months. The mean time to presentation was 7.0 (± 9.2) hours, and 9/42 (21%) children had a prior history of RHS. The majority of patients (35/42, 83%, 95% confidence interval (CI) 69-92%) had a normal elbow ultrasound. 6/42 (14%, 95% CI 6-28%) patients had an elevated PFP and 2/42 (5%, 95% CI 0.5-17%) had LH. Clinical reduction was successful in 100% of patients, and there were no complications reported on follow-up.

## Conclusion

The majority of children with RHS have a normal PFP on elbow ultrasound, but elevated PFP and LH are possible findings. Reduction maneuvers for RHS should be attempted in the setting of a normal elbow ultrasound when RHS or elbow fracture is uncertain, but with abnormal ultrasound findings, X-rays may be considered.

